# Comparative meta-analysis of antimicrobial resistance from different food sources along with one health approach in the Egypt and UK

**DOI:** 10.1186/s12866-023-03030-5

**Published:** 2023-10-16

**Authors:** Gunjan, Himanshu, Riya Mukherjee, Jasmina Vidic, Marisa Manzano, Elcio Leal, V. Samuel Raj, Ramendra Pati Pandey, Chung-Ming Chang

**Affiliations:** 1 Graduate Institute of Biomedical Sciences, Chang Gung University, No. 259, Wenhua 1St Road, Guishan Dist, Taoyuan City, 33302 Taiwan; 2 Master & Ph.D. Program in Biotechnology Industry, Chang Gung University, No. 259, Wenhua 1St Road, Guishan Dist, Taoyuan City, 33302 Taiwan; 3 Université Paris-Saclay, Micalis Institute, INRAE, AgroParisTech, 78350 Jouy-en-Josas, France; 4 Department of Agriculture Food Environmental and Animal Sciences, University of Udine, 33100 Udine, Italy; 5 Laboratório de Diversidade Viral, Instituto de Ciências Biológicas, Universidade Federal Do Pará, Belem, Pará, 66075-000 Brazil; 6 School of Health Sciences and Technology (SoHST), UPES, Bidholi, Dehradun, 248007 Uttarakhand India; 7 Department of Medical Biotechnology and Laboratory Science, Chang Gung University, No. 259, Wenhua 1St Road, Guishan Dist, Taoyuan City, 33302 Taiwan; 8 Laboratory Animal Center, Chang Gung University, No. 259, Wenhua 1St Road, Guishan Dist, Taoyuan City, 33302 Taiwan

**Keywords:** Antibiotics, Antimicrobial resistance (AMR), Multi-drug resistant (MDR), Antimicrobial stewardship, One Health (OH)

## Abstract

**Background:**

Antimicrobial resistance (AMR) is a critical global issue that poses significant threats to human health, animal welfare, and the environment. With the increasing emergence of resistant microorganisms, the effectiveness of current antimicrobial medicines against common infections is diminishing. This study aims to conduct a competitive meta-analysis of surveillance data on resistant microorganisms and their antimicrobial resistance patterns in two countries, Egypt and the United Kingdom (UK).

**Methods:**

Data for this study were obtained from published reports spanning the period from 2013 to 2022. In Egypt and the UK, a total of 9,751 and 10,602 food samples were analyzed, respectively. Among these samples, 3,205 (32.87%) in Egypt and 4,447 (41.94%) in the UK were found to contain AMR bacteria.

**Results:**

In Egypt, the predominant resistance was observed against β-lactam and aminoglycosides, while in the United Kingdom, most isolates exhibited resistance to tetracycline and β-lactam. The findings from the analysis underscore the increasing prevalence of AMR in certain microorganisms, raising concerns about the development of multidrug resistance.

**Conclusion:**

This meta-analysis sheds light on the escalating AMR problem associated with certain microorganisms that pose a higher risk of multidrug resistance development. The significance of implementing One Health AMR surveillance is emphasized to bridge knowledge gaps and facilitate accurate AMR risk assessments, ensuring consumer safety. Urgent actions are needed on a global scale to combat AMR and preserve the effectiveness of antimicrobial treatments for the well-being of all living beings.

## Highlights

1. Egypt and the United Kingdom (UK) have the highest prevalence rates of *E. coli.*

2. Chicken has the highest prevalence of microbial resistance strains.

3. Egypt has the highest rate of β-lactam and aminoglycoside resistance.

4. UK has the highest rate of tetracycline and β-lactam resistance.

5. Resistance to multiple drugs has been identified in both nations.

## Introduction

Antibiotic resistance (AMR) has been identified as one of the biggest global health concerns to people and animals, affecting not only developed and developing nations but throughout the world. It is spreading across the globe due to pathogenic bacteria. AMR is a concern, regardless of geographical boundaries, healthcare knowledge levels, or national economic status [1]. According to the United Kingdom (UK) Government-commissioned Review on AMR, Current estimates put the annual global mortality rate from AMR at 700,000. By 2050, this figure could potentially reach a staggering 10 million annual fatalities [2–4]. Estimates suggest that AMR is responsible for 25,000 annual deaths in the EU alone [5]. According to a recent article, in 2019, bacterial AMR directly caused 1.27 million deaths and was additionally associated with over twice that number of fatalities. In 2019, antibiotic-resistant Escherichia coli alone resulted in the deaths of nearly 200,000 people [6]. Most of these fatalities are expected to occur in underdeveloped nations.AMR will also have a negative impact on the world economy and hinder development efforts. It is estimated that failing to address AMR until 2050 will cost $100 trillion in total losses. The global GDP may decline by as much as 3.5%. Most of the developing world's poorest nations will experience significant economic losses [7]. The latest data published by the UK Health Security Agency (UKHSA) [8] reveals that the estimated total number of serious antibiotic resistant infections in England rose by 2.2% in 2021 compared to 2020. While in Egypt, a total of 4.95 million individuals died in 2019 were afflicted with drug-resistant infections. Among these cases, 1.27 million deaths were directly attributed to AMR [9].

**Fig. 1:**
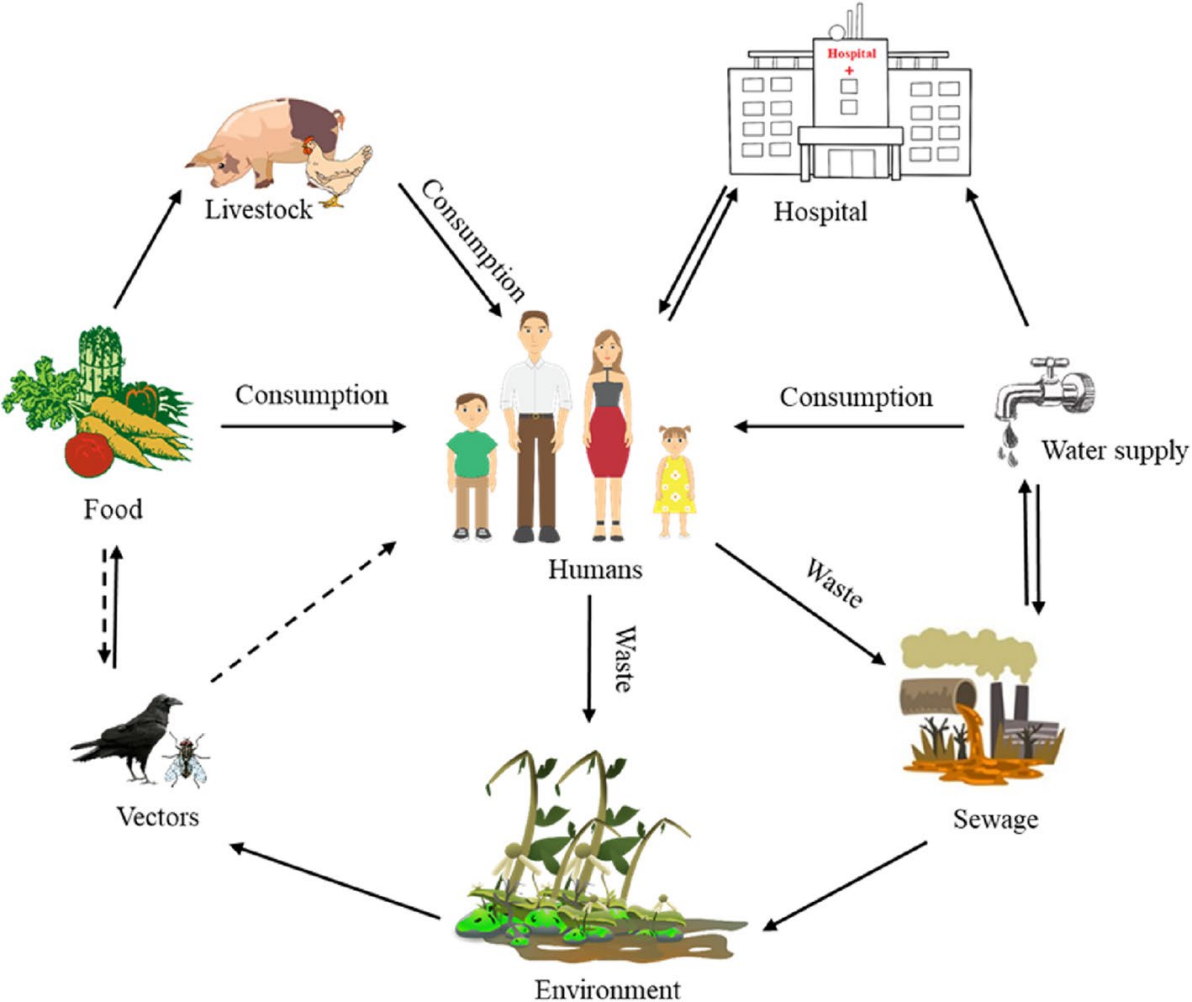
Interconnections of complex AMR amongst several health sectors. A schematic diagram for the complex transmission pathways of AMR genes and drug-resistant bacteria between human, livestock and environmental reservoirs. The dashed lines represent potential transmission pathways [15]. (Reference?)

Beside humans, billions of pets, livestock, and fish rely on these drugs, as curative or preventive medicines, or as questionable growth boosters. However, every time we use antibiotics, we put bacteria under selection pressure to modify or transfer pieces of DNA, potentially leading to drug resistance [10]. As a result, the misuse and overuse of antibiotics are significant contributors to the resistance phenomena, along with other local and global variables that promote the spread of resistant bacteria and their genes [11]. AMR is characterized by complex interactions involving many microbial populations. With this complexity and ecological nature in perspective, it becomes important to address the resistance issue via a coordinated, multi-sectorial strategy, such as One Health [12–14], as depicted in Fig. 1.

One Health is defined as "the collaborative approach of numerous health science professionals, as well as their allied disciplines and institutions, working locally, nationally, and internationally to achieve optimal health for humans, livestock, biodiversity, flora, and our ecosystem." by the World health organization (WHO) [16] and the Food and Agriculture Organization of the United Nations (FAO) [17, 18]. This comprehensive viewpoint makes it possible to comprehend how human interactions with the environment influence the spread and occurrence of diseases as well as how society may be prone to the potential burden of these diseases [19]. Several International organizations, such as the WHO, FAO and World Organization for Animal Health (OIE), have a significant impact on monitoring antibiotic consumption providing the necessary information to combat AMR. The WHO has unveiled several international measures to address AMR at the global level such as “Global Action Plan (GAP)”, “Global Antimicrobial Resistance and Use Surveillance System (GLASS) [20–22] and locally “Egypt’s National Action Plan on Antibiotic Resistance”, “Central Asian and European surveillance of AMR (CAESAR)”, “UK’s National Action Plan”, “European Surveillance of Antimicrobial Consumption Network”, “European Antimicrobial Resistance Surveillance Network (EARS-Net)” and “Joint Programming Initiative on AMR (JPIAMR)”. These programmes focus on the One Health concept, which links human and animal health to their respective ecosystems (Table 3).

The major carriers of AMR in the UK are raw and undercooked poultry [23]. From farm to product, there are numerous processes involved in producing pork, meat, and poultry, including breeding and finishing, animal transportation, slaughter, cutting, processing, and packing. Each of these processes might be a source of bacterial contamination [24, 25]. While meat is the primary source of protein in Egypt, raw milk is also believed to contribute significantly to the development of various health issues [26, 27]. Additionally, due to the poor quality, Egyptian street food, particularly meat products, may pose a risk. The following factors, including raw material use, inadequate worker cleanliness, and holding for a long time cause food to become contaminated with pathogenic bacteria [28].

The rising prevalence of bacteria in various food sources contributes to an escalation in drug resistance and affects the susceptibility of infections to antibiotics. It is important to determine the burden based on both of these counterfactual scenarios because we do not know the amount to which drug-resistant infections would be replaced by susceptible infections or by no infections in a scenario in which all drug resistance was removed. In this study, a systematic research and meta-analysis on AMR present in food samples were conducted in an African country Egypt (developing) and a European country UK (highly developed), using data published on Medline search engine between 2013–2022.

## Materials and methods

### Search methodology

**Fig. 2:**
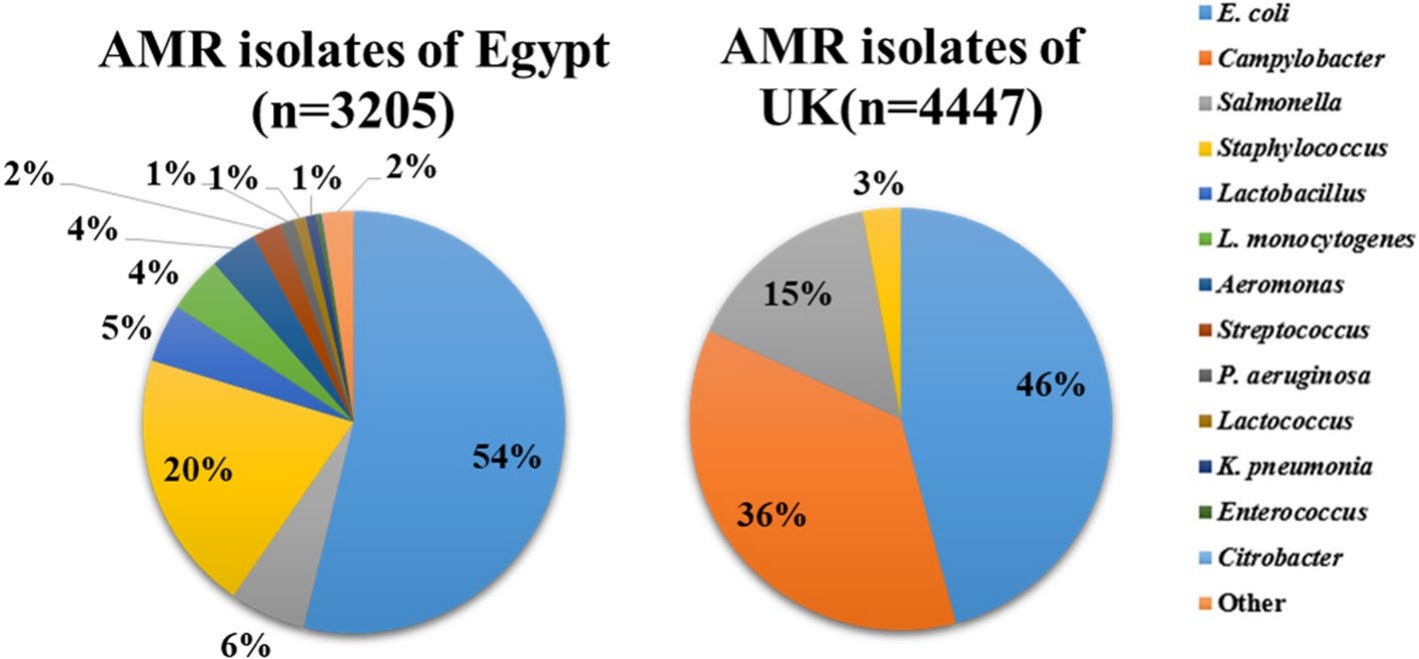
Comparative analysis of AMR Isolates of Egypt (*n* = 3205) and UK (*n* = 4447)

Data from different Medline search engines, including PubMed (https://pubmed.ncbi.nlm.nih.gov/), Google Scholar (https://scholar.google.com/), and Science Direct (https://www.sciencedirect.com/), were examine to find relevant publications that had been published between January 2013 and December 2022. The papers that are suggested for reporting systematic reviews and meta-analyses. Preferred Reporting Items for Systematic Reviews and Meta-Analysis (PRISMA) guideline (http://www.prisma-statement.org/) was followed to obtain the data, and the pertinent medical subject heading (MeSH) word was also used to retrieve the data provided below. For e.g., “Pathogen transfer from different food sources along with antimicrobial resistance”, “AMR Spreading from Different pathogens along with One Health”, “Multi drug resistance”, “Drug susceptibility tests of different pathogens isolated from different food sources”, “AMR assessment method”, “MDR”, “Egypt”, “United Kingdom” “UK” “One Health” “surveillance” are the keywords as well as the MeSH terms that were used. The search queries were applied using the Boolean operators "AND" and "OR." Fig. 2 depicts the search strategy for the PubMed/MEDLINE database in accordance with PRISMA guidelines.

### Selection criteria

A total of 49 papers were selected for retrieval of information and for the inclusion or omission of data. The following was the basis for the studies that mainly composed the meta-analysis: (i) accessibility of the article's full text and abstract; (ii) studies with author names, publication year, region, overall number of isolates, total samples collected, and circumstances; (iii) observations of food pathogens and AMR; (iv) mention of the pathogen analysis method; (v) mention of sample sources (such as animal and vegetable food origins, dairy products, environmental samples, food handlers, slaughterhouses, etc.); and (vi) an AMR evaluation approach that takes into account several molecular methodologies.

### Exclusion criteria

Studies were disqualified if they (a) were not review articles (b) proper screening techniques were not mentioned (c) did not have any book chapters included, (d) essential statistics were missing (e) AMR was not analyzed, and (f) were not conducted in Egypt and UK (g) did not have any publications published between 2013 and 2022.

### Data extraction and quality assessment

To establish a baseline, full versions of allegedly relevant articles were obtained. The author names, publication year, geography, the total number of isolates, and total samples from each article were collected independently and recorded on a spreadsheet (Microsoft Excel® 2013) for pre-testing prior to full extraction. Text, tables, and figures were used to extract the data. The results were examined, and a pie chart was used to show the AMR data and the cited articles using Mendeley (version 1.19.8).

### Food category

The food categories considered were beef, chicken, retail chicken, raw meat, ready-to-eat (RTE) meat, fish, milk, other dairy product (cheese, ice cream, yoghurt, cream), water, vegetables, animals (pig, sheep, dog, badger, fox), and environmental samples (water, drainages) shown in Tables 1 and 2.

**Table 1:** Prevalence of different microorganisms in Egypt's food resources from 2013—2022

Microbes	Beef Total *n* = 710	Chicken Total *n* = 3175	Raw meat Total *n* = 985	RTE food Total *n* = 575	Fish Total *n* = 560	Milk Total *n* = 1591	Other dairy products Total *n* = 1443	Vegetables Total *n* = 539	Water Total *n* = 173	Total *n* = 9751
	**n and** **(%)**	**n and** **(%)**	**n and** **(%)**	**n and** **(%)**	**n and** **(%)**	**n and** **(%)**	**n and** **(%)**	**n and** **(%)**	**n and** **(%)**	**n and** **(%)**
***E. coli***	90 (12.68)	1087(34.24)	177(17.97)	5(0.87)	-	204(12.82)	118(8.18)	-	44(25.43)	1725(17.69)
***Staphylococcus***	-	-	44(4.47)	187(32.52)	-	158(9.93)	154(10.67)	-	-	643(6.59)
***Salmonella* spp.**	76(10.70)	103(3.24)	-	8(1.39)	-	-	-	-	-	187(1.92)
***Lactobacillus***	-	-	-	-	-	51(3.21)	93(6.44)	-	-	144(1.48)
***L. monocytogenes***	20(2.82)	-	18(1.83)	-	5(0.89)	18(1.13)	26(1.80)	51(9.46)	-	138(1.42)
***Aeromonas* spp.**	-	-	-	-	115(20.54)	-	-	-	-	115(1.18)
***Streptococcus***	-	-	-	-	7(1.25)	8(0.50)	57(3.95)	-	-	72(0.74)
***P. aeruginosa***	14(1.97)	-	-	-	-	-	20(1.39)	-	-	34(0.35)
***Lactococcus***	-	-	-	-	2(0.36)	7(0.44)	20(1.39)	-	-	29(0.30)
***K. pneumoniae***	4(0.56)	2(0.06)	-	-	-	-	20(1.39)	-	-	26(0.27)
***Enterococcus* spp.**	-	5(0.16)	-	-	8(1.43)	0(0)	-	-	-	13(0.13)
***Citrobacter* spp.**	-	2(0.06)	-	-	-	-	-	-	-	2(0.02)
**Other**	-	-	-	39(6.78)	-	38(2.39)	-	-	-	77(0.79)
**Total**	204(28.73)	1315(41.42)	239(24.26)	239(41.57)	137(24.46)	484(30.42)	608(42.13)	51(9.46)	44(25.43)	3205(32.87)

*N* Number of isolates; (%) of isolates

**Table 2:** Prevalence of different microorganisms in UK's food resources from 2013—2022

**Microbes**	**Chicken** **Total *n* = 919**	**Retail chicken** **Total *n* = 1530**	**Pig** **Total *n* = 1116**	**Raw meat** **Total *n* = 3959**	**Animals** **Total *n* = 1771**	**Dairy** **Total *n* = 300**	**Environmental** **Total *n* = 1007**	**Total** ***N* = 10,602**
	**n and** **(%)**	**n and** **(%)**	**n and** **(%)**	**n and** **(%)**	**n and** **(%)**	**n and** **(%)**	**n and** **(%)**	**n and** **(%)**
***E. coli***	415(45.16)	-	595(53.32)	-	901(50.88)	126(42)	-	2037(19.21)
***Campylobacter***	293(31.88)	1023(66.86)	-	285(7.20)	-	-	2(0.20)	1603(15.12)
***Salmonella***	-	-	101(9.05)	-	14(0.79)	145(48.33)	416(41.31)	676(6.38)
***Staphylococci***	-	-	-	-	131(7.40)	-	-	131(1.24)
**Total**	824(89.66)	1023(66.86)	696(62.37)	285(7.20)	1046(59.06)	271(90.33)	418(41.51)	4447(41.94)

*N* Number of isolates; (%) of isolates

## Result

### Identification of studies

The initial search strategy approach yielded 506 citations from electronic databases. After 225 duplicates were deleted, 281 records were evaluated for title and abstract. Out of these, 281 full-text papers were assessed, and in the initial systematic search, 133 records were excluded; 79 due to improper screening of data, 15 for no relevant interventions in animals to reduce antibiotics use, 37 were due to being review articles on MDR and 2 due to full access which could not be achieved. Of these 148 full-text articles, 98 were again excluded, of which 54 were excluded because they did not address AMR in the results, 32 were excluded due to irreverent outcomes, and 12 were excluded due to missing essential statistics. This systematic review and meta-analysis included 50 articles in total. The entire set of criteria for inclusion and exclusion are shown in Fig. 2.

### One health and AMR training programs in Egypt

Several international organizations such as the World Organization for Animal Health (OIE), World Health Organization (WHO) and Food and Agriculture Organization of the United Nations (FAO), have taken significant steps to address antibiotic resistance. These measures encompass the adoption of various plans, such as the National Action Plan on Antibiotic Resistance in 2018–2024 and [29, 30] the Global Action Plan on Antimicrobial Resistance-WHO [31]. These initiatives focus on infection prevention and control, AMR surveillance and management supported by the antimicrobial stewardship program, increasing public awareness, and investing in novel medications. Measures to build national capacity were identified, along with a variety of other interventions [32]. It will make it possible to examine the relationship between AMR and antimicrobial use in many contexts (including those involving animals, people, and the environment) and to evaluate the impact of interventions within and across sectors [1, 33, 34]. An additional operational initiative is the Global Antimicrobial Resistance and Use Surveillance System (GLASS) [21], It promotes nations to adopt surveillance methods based on systems that include epidemiological, clinical, and population-level data rather than only laboratory data and fosters the development of the AMR evidence base (Table 3).

**Table 3:** Antimicrobial resistance monitoring and vigilance in Egypt and the UK

Country	One health programs	Significance
**Egypt**	National Action Plan on Antibiotic Resistance https://www.who.int/publications/m/item/egypt-national-action-plan-for-antimicrobial-resistance Accessed 28 January 2022	Control AMR by raising public health awareness, strengthen infection control measures, containment of the emergence and spread of AMR organisms, activate Lab-based surveillance system, rational use of antimicrobials and finding novel therapies
	Global Action Plan (GAP) https://www.who.int/publications/i/item/9789241509763	Infection prevention and control, AMR surveillance and management supported by the antimicrobial stewardship program, raising public awareness, investing in new medicines, and a variety of other interventions
	Global Antimicrobial Resistance and Use Surveillance System (GLASS) https://www.paho.org/en/documents/global-antimicrobial-resistance-and-use-surveillance-system-glass-report-2022	It promotes nations to adopt surveillance methods based on systems that include epidemiological, clinical, and population-level data rather than only laboratory data and fosters the development of the AMR evidence
**UK**	UK’s National Action Plan https://www.ecdc.europa.eu/en/about-us/partnerships-and-networks/disease-and-laboratory-networks/esac-net	It sets out commitments in line with the Open Government Partnership values of access to information, civic involvement, public accountability, and technology and innovation
	WHO’s Global Antimicrobial Resistance Surveillance System (GLASS) https://www.who.int/initiatives/glass	Provides a standardized approach for countries to collect, analyses, and share AMR data, with the goal of supporting capacity development and monitoring the status of existing or newly formed national AMR surveillance systems
	European Antimicrobial Resistance Surveillance Network (EARS-Net) https://www.ecdc.europa.eu/	Evaluate the overall comparability of routinely collected test results and assess the accuracy of quantitative antimicrobial susceptibility test results

### One health and AMR training programs in United Kingdom (UK)

Several international organizations such as the WHO, OIE and FAO adopt several plans such as UK’s National Action Plan 2019–2024, which is a national action plan to combat AMR both within and beyond our borders. Developed in collaboration with a diverse variety of partners from various sectors [22], another is WHO’s Global Antimicrobial Resistance Surveillance System (GLASS) which is an international cooperative effort to standardize AMR surveillance that was started to advance knowledge through monitoring and investigation [35]. European Surveillance of Antimicrobial Consumption Network, European Antimicrobial Resistance Surveillance Network (EARS-Net) evaluates the overall comparability of routinely collected test results and assess the accuracy of quantitative antimicrobial susceptibility test results [34]. Furthermore, the Central Asian and European Surveillance of Antimicrobial Resistance network (CAESAR) serves as a network of encompassing national antimicrobial resistance (AMR) surveillance systems that includes all WHO European Region countries that are not members of the European Antimicrobial Resistance Surveillance Network (EARS-Net), which is coordinated by the European Union's European Centre for Disease Prevention and Control [36]. Additionally, the EU established the Joint Programming Initiative on AMR (JPIAMR), aims to better coordinate global AMR research efforts (Table 3).

### Comparative meta-analysis

Out of 506 eligible papers, as indicated in Fig. 2, 50 publications (34 from Egypt and 16 from the UK) were included in the meta-analysis and systematic study. A total of 7,652 AMR tests (3,205 from Egypt and 4447 samples from the UK) were obtained for various bacteria found in various food items, animals, and environments. A total of 12 different antimicrobial agents/drugs and 13 different bacteria showedresistance. The sample sources, species of bacteria, and their proportions are represented in Tables 1 and 2.

#### Comparative meta-analysis in Egypt

Out of 220 eligible studies related to pathogens carrying AMR in Egypt from 2013 to 2022, 34 full-text articles were included for further examination [37–70]. Out of 9,751 samples, 3205 (32.87%) found positive prevalence of which samples included beef 204 (28.73%), chicken 1315 (41.42%), raw meat 239 (24.26%), ready to eat food 239 (41.57%), fish 137 (24.46%), milk 484 (30.42%), other dairy products 608 (42.13%), vegetables 51 (9.46%), and water 44 (25.43%) showed positive prevalence for various pathogens including *E. coli* 1725 (17.69%), *Staphylococcus* 643 (6.59%), *Salmonella* spp. 187 (1.92%), *Lactobacillus* 144 (1.48%), *L. monocytogenes* 138 (1.42%), *Aeromonas* spp. 115 (1.18%), *Streptococcus* 72 (0.74%), *P. aeruginosa* 34 (0.35%), *Lactococcus* 29 (0.30%), *K. pneumonia* 26 (0.27%), *Enterococcus* spp. 13 (0.13%), *Citrobacter* spp. 2 (0.02%) and other 77 (0.79%) (Table 1). It can be assumed that *E. coli* and *Staphylococcus* are the most prevalent in Egypt. Approximately 34% *E. coli* isolates in Egypt originated from chicken and 25% from water. Furthermore, it was found that *L. monocytogenes* is the only species that was found in vegetables only. *Listeria monocytogenes* is the causative agent of listeriosis and a serious threat to the health of certain populations, including the elderly, immunocompromised people, and pregnant women. It is an uncommon foodborne disease with a 20%-30% death rate. *L. monocytogenes* is also common in the environment and can infect food-processing settings, posing a threat to the food chain [71, 72].

#### Comparative meta-analysis in UK

In a comprehensive analysis of 286 research papers pertaining to foodborne pathogens in the UK between 2013 and 2022, only 16 full-text articles were selected for meta-analysis, as referenced in sources [15, 21, 71–83]. Within a dataset comprising 10,602 samples, it was observed that 41.94% of these samples exhibited a positive prevalence of pathogens. Notably, chicken 824 (89.66%), retail chicken 1023 (66.86%), and dairy products 271 (90.33%) displayed particularly high positive prevalence rates, while other sources such as pigs 696 (62.37%), raw meat 285 (7.20%), animals 1046 (59.06%), and the environment 418 (41.51%) also demonstrated varying degrees of pathogen prevalence. Key pathogens identified included *E. coli.* 2037 (19.21%), *Campylobacter* 1603 (15.12%), *Salmonella* 676 (6.38%), and *Staphylococci* 131 (1.24%) (Table 2). It can be assumed that *E. coli* and *Campylobacter* are the most prevalent in the UK. About 53% of *E. coli* isolates in the UK originated from pigs. Additionally, it was found that *Campylobacter* is the only pathogen i.e., prevalent in retail chicken and raw meat only. It is one of the most prevalent causes of bacterial diarrheal sickness globally, including acute enteritis, extra intestinal infections (for example, bacteremia, abscess, and meningitis), and post infectious complications. In most cases, *Campylobacter* causes a self-limiting clinical illness that lasts 5 to 7 days; the infection resolves without antimicrobial therapy in the vast majority of cases, although 5% to 10% of individuals experience a recurrence after their initial illness [73].

### Comparison of AMR isolates in Egypt and UK and their antimicrobial resistance

**Fig. 3:**
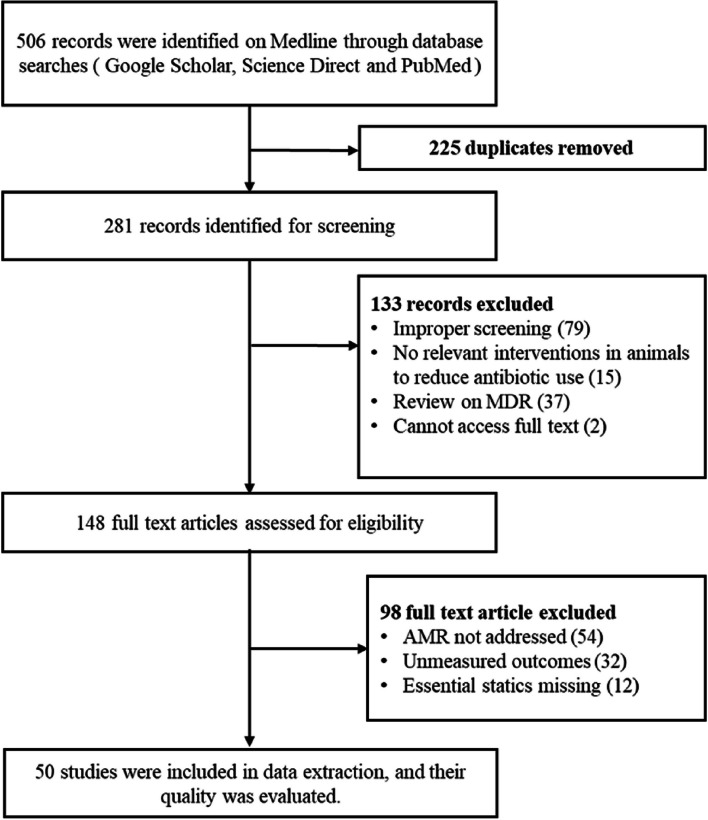
Diagram showing the flow of the study selection process according to Preferred Reporting Items for Systematic Reviews and Meta-Analyses (PRISMA) [84]

A comparison of their AMR isolates in Egypt and UK is provided in Fig. 3, indicating a similar incidence of *E. coli* in both nations, representing about 54% in Egypt and 46% in the UK. Similarly, *Campylobacter* is the second most common pathogen in the UK, accounting for 36%, however in the Egypt *Staphylococcus* is the second most prevalent pathogen with 19%. *Staphylococcus* abundance in Egypt which is mostly associated with high intake of raw and RTE meat, milk, and other dairy items, but *Campylobacter* prevalence in the UK is solely related to vegetable consumption.

**Fig. 4:**
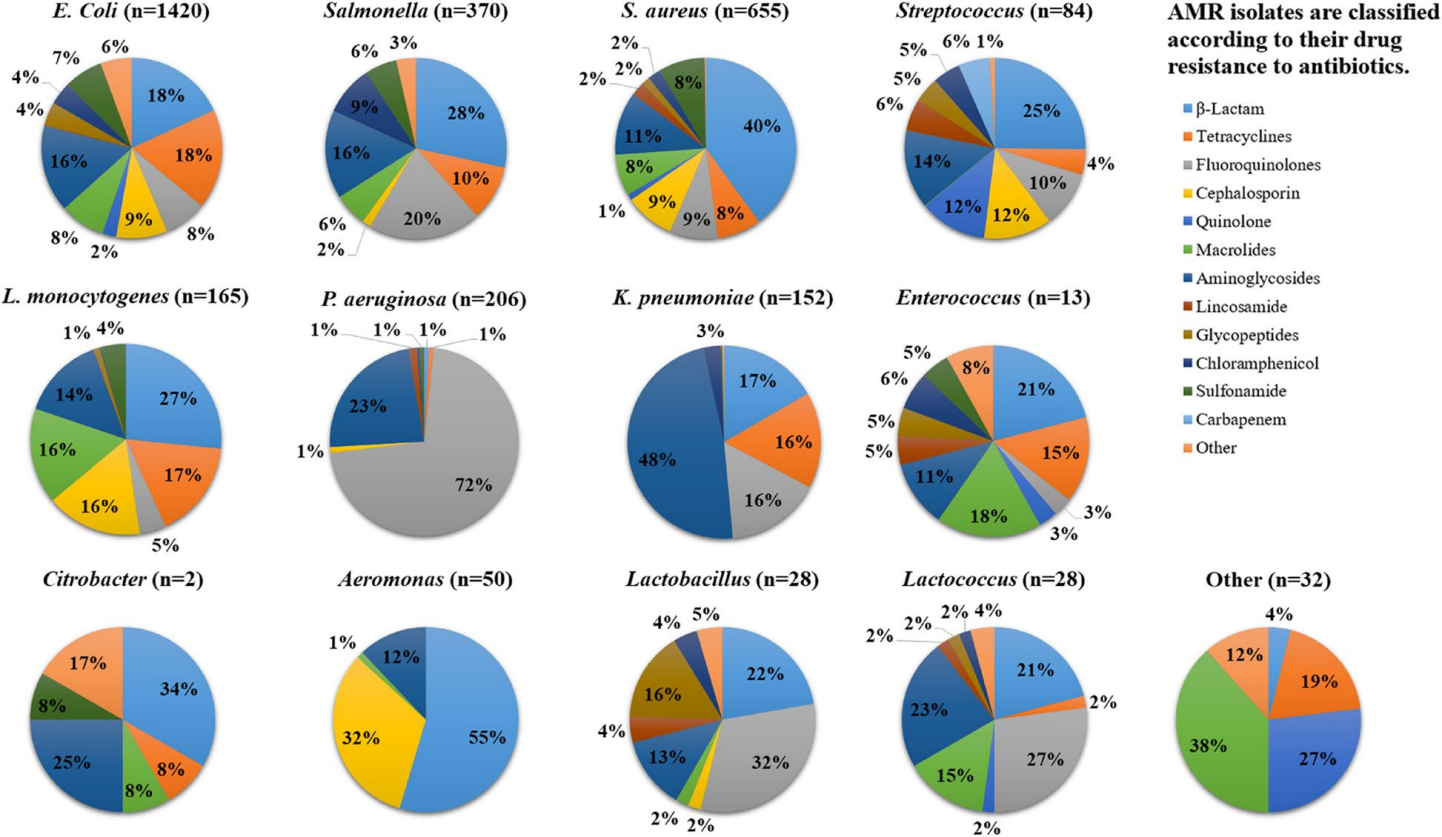
AMR positive isolates from different food sources (*n* = 2883) in Egypt, (n represent the number of isolates) from 2013 to 2022

**Fig. 5:**
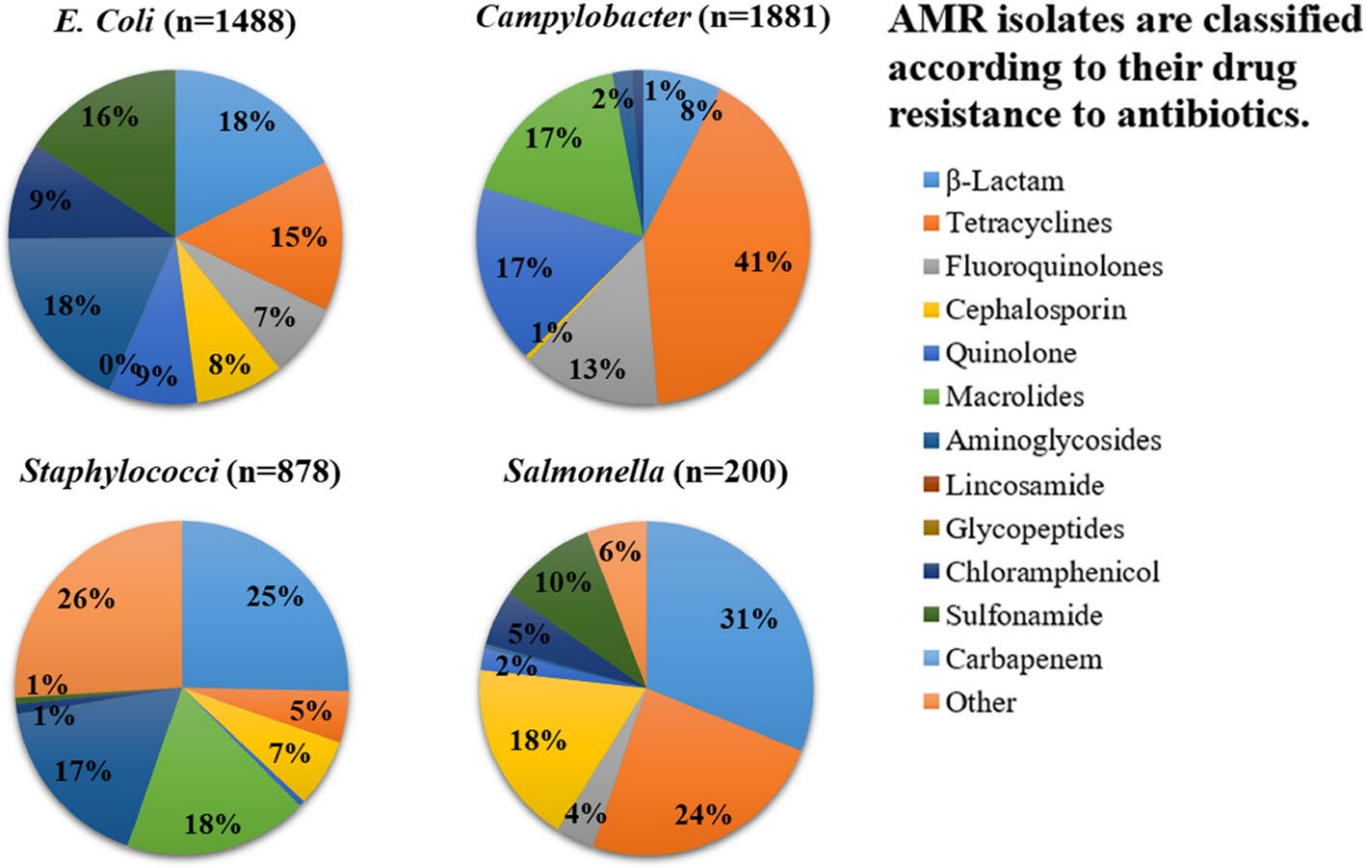
AMR positive isolates from different food sources (*n* = 4563) in UK, (n represent the number of isolates) from 2013 to 2022

The analyses of antimicrobial agents found and antibiotic resistances observed for each pathogen isolate in Egypt and the UK are illustrated in Figs. 4 and 5. *E. coli* with multiple drug resistance was observed in both Egypt and the UK, as well as MDR strains of *Staphylococcus* spp., *Salmonella*, and *K. pneumonia* in Egypt and *Staphylococcus* spp., and *Campylobacter* spp. in the UK. Figure 4 shows that the majority of the bacteria found in Egypt exhibited microbial resistance to β-lactams and aminoglycosides, followed by fluoroquinolones and tetracyclines, whereas the majority of the bacteria found in the UK showed microbial resistance to tetracyclines and β-lactams, followed by aminoglycosides and sulfonamide (Fig. 5). It is concerning because the majority of the microorganisms reported were multidrug resistant.

## Discussion

This study provides a comprehensive assessment of the abundance of foodborne pathogens and the presence of AMR genes in Egypt and the UK. With the goal of establishing a link between the prevalence of AMR and various bacteria, data published over the last ten years (2013 to 2022) were examined. 56 papers were selected and met the inclusion criterion out of 190 papers. Beef, chicken, retail chicken, raw meat, ready to eat meat, fish, milk, other dairy products (cheese, ice cream, yoghurt, cream), water, vegetables, pigs, animals (sheep, dog, badger, fox), environmental samples (water, drainages) and other food sources were all subjected to a planned follow-up AMR risk group evaluation.

The meta-analysis suggested that foods were highly contaminated with *E.coli*, and *Staphylococcus* spp. in Egypt, while *Campylobacter* spp. was the prevalent bacterium detected in food in the UK. The obtained data reflected that contamination occurred in the food mostly eaten in each country. The isolated bacteria showed resistance mainly to β-Lactam, tetracyclines, fluoroquinolones, cephalosporin, quinolone, macrolides, aminoglycosides, lincosamide, amphenicols, Glycopeptides, Chloramphenicol, sulfonamide, carbapenem, and other. The wide range of resistance genes shows that strains derived from food may have an impact on the environment, animals, and humans.

In Egypt, out of a total of 20,353 isolates, 9,751 (48%) were found to be positive for AMR. Among these, 3,205 (16%) of the isolated bacteria exhibited resistance to β-lactams and aminoglycosides.The resistance to fluroquinolone, tetracycline, cephalosporin, macrolides and sulfonamide had middle prevalence whereas chloramphenicol, glycopeptides, quinolones, lincosamide and carbapenem showed low prevalence. Whereas in the UK, out of 20,353 isolates, 10,602 (52%) samples were positive for AMR by which 4447 (22%) bacteria were isolates. Tetracycline, and β-lactam showed the highest resistance occurrence, while aminoglycosides, sulfonamide and quinolones showed middle prevalence and chloramphenicol, cephalosporin, fluoroquinolones and macrolides showed low prevalence.

There was a noticeable difference in antimicrobial susceptibility patterns between the groups. This difference in AMR may be due to the antimicrobial drugs not only used to treat various infections in animals but to inhibit the growth of bacteria [74–76]. The Food Standards Agency (FSA) in the UK is responsible for ensuring food safety and hygiene in England, Wales, and Northern Ireland. It collaborates with local authorities to enforce food safety requirements, and its employees work in meat plants to ensure that criteria are met [77]. However, the Egyptian National Food Safety Authority’s (NFSA) goal is to protect Egyptian customers' health and safety by imposing minimum requirement standards for food exported to Egypt [78]. Among the different sources and transmission pathways examined, faecal fertilizers, irrigation, and surface water were discovered to contribute the most to AMR. Raw foods are regarded substantially risky to consumers because resistant microbes can thrive in untreated food [79, 80].

Today's health concerns are usually complex, transboundary, multifactorial, and cross-species, and it is unlikely that sustainable mitigation methods would be developed if handled just from a medical, veterinary, or ecological perspective [81, 82]. To better understand the complexity of the situation there is a need to implement One Health approach to a variety of sectors as well as the larger topic of antibiotic resistance at the animal-human–environment interface [83]. Therefor it is important to understand the top pathogen-drug combinations contributing to the burden of bacterial AMR trends worldwide, and the present severity of the problem. If AMR continues to progress without restraint, numerous bacterial pathogens could potentially become significantly more lethal in the future than they are at present.

## Conclusion

This comprehensive systematic review and meta-analysis provided a summary of the current state of AMR in Egypt and the UK from the aspect of One Health. The levels of AMR reported in Egypt between 2013 and 2022 are of concern, especially regarding ancient antibacterial agents such as β-Lactam, 1st and 2nd generation cephalosporin, Aminoglycoside or tetracycline. The high levels of AMR and the identification of pertinent levels of other agent resistance Tetracycline, β-Lactam, Aminoglycosides, 3rd and 4th generation cephalosporin or fluoroquinolones, as well as the detected resistance to these drugs in both Egypt and the UK point to the necessity of enacting effective controls regarding access to antibacterial agents as well as the creation of educational campaigns to raise public awareness of the importance of prudent use of antibacterial agents.

The unusual number of isolates especially most dangerous pathogenic bacteria such as *E. coli*, *Klebsiella* spp., *Streptococcus* spp., *Staphylococcus* spp., *Salmonella*, *Campylobacter* spp. found on foodstuffs exhibiting intermediate levels of resistance to multiple antimicrobials highlights the necessity for a One Health approach to overcoming the impending pandemic. The success of the OH strategy is dependent not just on local initiatives, but also on socio-culture, socioeconomic, and institutional initiatives at an institutionalized and systemic level.

## Data Availability

The authors confirm that the data supporting the findings of this study are available within the article.
